# The genome sequence of the red ripple bryozoan, *Watersipora subatra *(Ortmann, 1890)

**DOI:** 10.12688/wellcomeopenres.22824.2

**Published:** 2026-02-11

**Authors:** John Bishop, Christine A. Wood, Marine Biological Association Genome Acquisition Lab, Darwin Tree of Life Barcoding collective, Wellcome Sanger Institute Tree of Life Management, Samples and Laboratory team, Wellcome Sanger Institute Scientific Operations: Sequencing Operations, Wellcome Sanger Institute Tree of Life Core Informatics team, Tree of Life Core Informatics collective, Darwin Tree of Life Consortium

**Affiliations:** 1 The Marine Biological Association, Plymouth, England, UK

**Keywords:** Watersipora subatra, red ripple bryozoan, genome sequence, chromosomal, Cheilostomatida

## Abstract

We present a genome assembly from an individual *Watersipora subatra* (the red ripple bryozoan; Bryozoa; Gymnolaemata; Cheilostomatida; Watersiporidae). The genome sequence spans 783.70 megabases. Most of the assembly is scaffolded into 11 chromosomal pseudomolecules. The mitochondrial genome has also been assembled and is 14.14 kilobases in length. Gene annotation of this assembly on Ensembl identified 16,835 protein-coding genes. This assembly was generated as part of the Darwin Tree of Life project, which produces reference genomes for eukaryotic species found in Britain and Ireland.

## Species taxonomy

Eukaryota; Opisthokonta; Metazoa; Eumetazoa; Bilateria; Protostomia; Spiralia; Lophotrochozoa; Bryozoa; Gymnolaemata; Cheilostomatida; Flustrina; Smittinoidea; Watersiporidae; *Watersipora*; *Watersipora subatra* (Ortmann, 1890) (NCBI:txid2589382).

## Background

The cheilostomatid bryozoan *Watersipora subatra* (Ortmann, 1890) grows across a solid surface as a colony of contiguous zooids (iterated ‘individuals’, *c.* 1.0 × 0.4 mm in size) that extends by recurrent budding of new zooids at the colony edge to create a continuous patch or sheet a single layer thick. Active peripheral parts of the colony are rich orange-red in colour, but older, inner regions become moribund and darken to blackish. Secondary patches of growth can arise within the darkened sections to grow over the existing colony surface or form raised, double-sided (back-to-back) orange-red lobes. The zooidal orifice (feeding opening) at the extreme distal end of the zooid is closed by a blackish operculum, so that the colony has a regular array of black dots when viewed close-up. The surface of the colony is relatively smooth and featureless because, unlike many species, the zooid lacks spines around the orifice and avicularia (non-feeding zooidal polymorphs), and an ovicell (separate brooding structure) is absent, the embryo being brooded within the zooidal chamber.

The taxonomy of the genus *Watersipora* is complex and has been the subject of much confusion ( Gauff *et al.*, 2023; Vieira *et al.*, 2014). Of 13 accepted species, four are currently spreading throughout the world, assisted by human activities. Of these, confusion between *W. subatra* and *W. subtorquata* has been particularly prevalent. Current understanding is that *W. subatra* is present in NW Europe, Norway, the Atlantic coast of the Iberian Peninsula, the western Mediterranean (French coast), Australia, New Zealand, Japan, Korea, California and Hawai’i ( Gauff *et al.*, 2023). *W. subatra* was first found in the UK in 2008 as *W. subtorquata* ( Ryland *et al.*, 2009). The species frequently fouls submerged surfaces in ports, harbours and marinas, but also encrusts stones/rocks, shells, holdfasts etc. low on the shore and in the shallow subtidal. On southern UK shores vertical, over-hung or downward-facing rock faces are particularly favoured places where *W. subatra* can achieve almost complete coverage.

Here we present the first chromosomally complete genome sequence for *Watersipora subatra*, based on a specimen from Plymouth, England, UK. This assembly is the first publicly available complete genome for the genus *Watersipora* and the family Watersiporidae as of February 2026, based on data retrieved via NCBI Datasets ( O’Leary *et al.*, 2024). This assembly was generated as part of the Darwin Tree of Life Project, which aims to generate high-quality reference genomes for all named eukaryotic species in Britain and Ireland to support research, conservation, and the sustainable use of biodiversity.

## Genome sequence report

**Figure 1.**
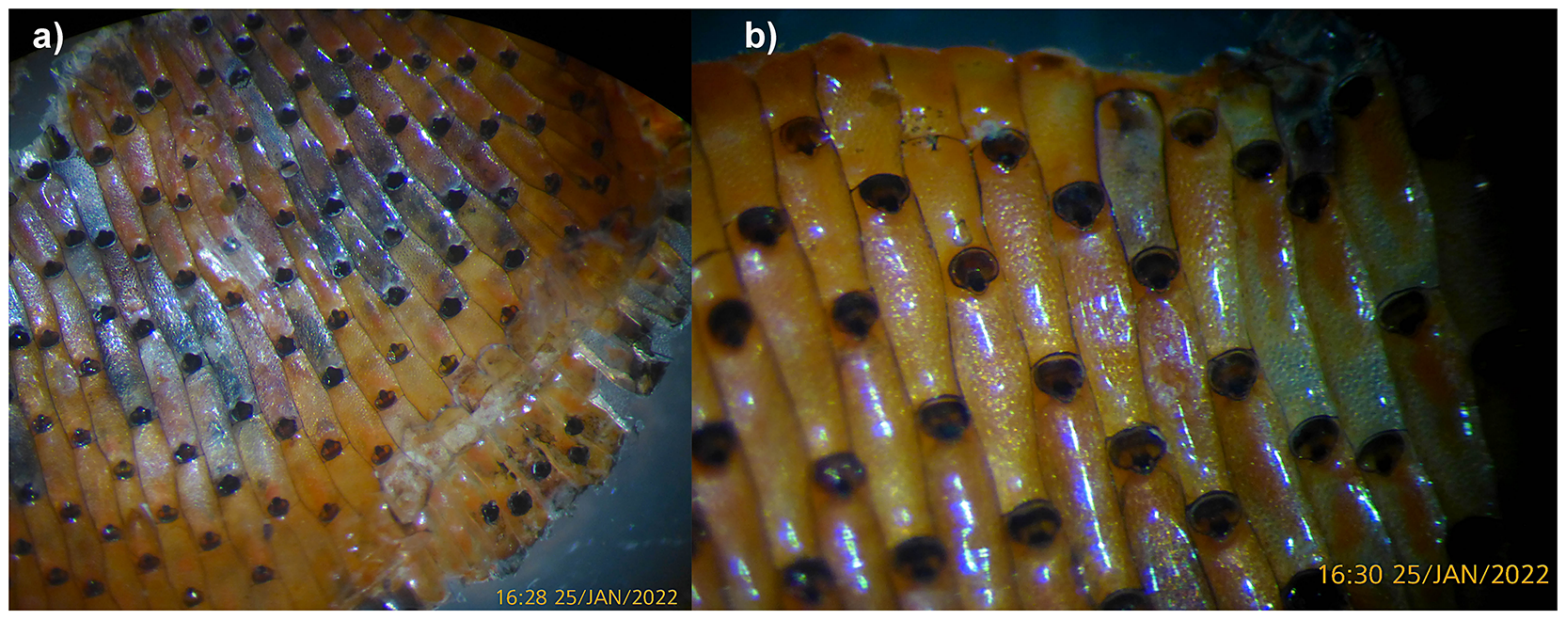
Photographs of the *Watersipora subatra* (tzWatSuba1) specimen used for genome sequencing.

The genome of an adult *Watersipora subatra* ( Figure 1) was sequenced using Pacific Biosciences single-molecule HiFi long reads, generating a total of 13.30 Gb (gigabases) from 2.20 million reads, providing approximately 39-fold coverage. Primary assembly contigs were scaffolded with chromosome conformation Hi-C data, which produced 131.99 Gb from 874.08 million reads, yielding an approximate coverage of 168-fold. Specimen and sequencing information is summarised in Table 1.

**Table 1.** Specimen and sequencing data for BioProject PRJEB62167.

Platform	PacBio HiFi	Hi-C	RNA-seq
**ToLID**	tzWatSuba1	tzWatSuba6	tzWatSuba4
**Specimen ID**	MBA-191001-001A	MBA-201019-018C	MBA-220119-006A
**BioSample (source individual)**	SAMEA7498434	SAMEA112148699	SAMEA13980352
**BioSample (tissue)**	SAMEA7500957	SAMEA112152773	SAMEA14013631
**Tissue**	modular colony	modular colony	modular colony
**Instrument**	Sequel IIe	Illumina NovaSeq 6000	Illumina NovaSeq 6000
**Run accessions**	ERR11458812; ERR11458813	ERR11468741	ERR11641139
**Read count total**	5.33 million	874.08 million	82.80 million
**Base count total**	30.93 Gb	131.99 Gb	12.50 Gb

**Figure 2.**
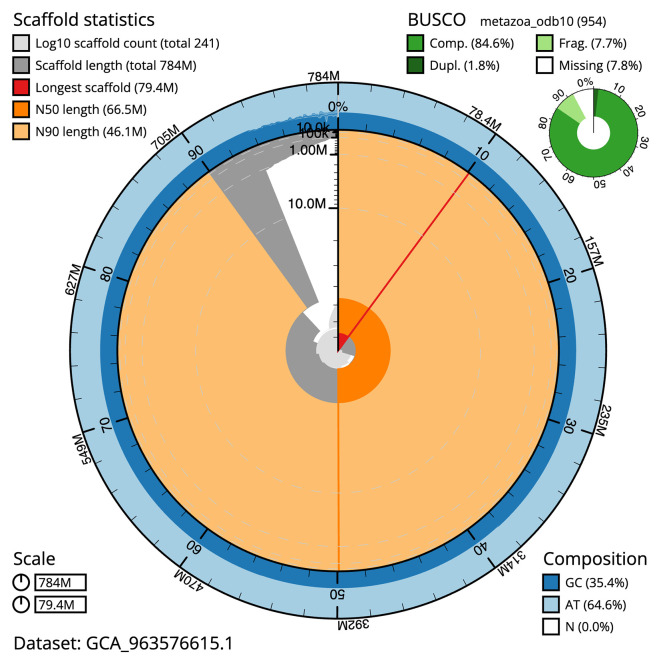
Genome assembly of *Watersipora subatra*, tzWatSuba1.1: metrics. The BlobToolKit snail plot shows N50 metrics and BUSCO gene completeness. The main plot is divided into 1,000 size-ordered bins around the circumference with each bin representing 0.1% of the 783,752,486 bp assembly. The distribution of scaffold lengths is shown in dark grey with the plot radius scaled to the longest scaffold present in the assembly (79,350,317 bp, shown in red). Orange and pale-orange arcs show the N50 and N90 scaffold lengths (66,545,008 and 46,061,466 bp), respectively. The pale grey spiral shows the cumulative scaffold count on a log scale with white scale lines showing successive orders of magnitude. The blue and pale-blue area around the outside of the plot shows the distribution of GC, AT and N percentages in the same bins as the inner plot. A summary of complete, fragmented, duplicated and missing BUSCO genes in the metazoa_odb10 set is shown in the top right. An interactive version of this figure is available at https://blobtoolkit.genomehubs.org/view/Watersipora_subatra/dataset/GCA_963576615.1/snail.

**Figure 3.**
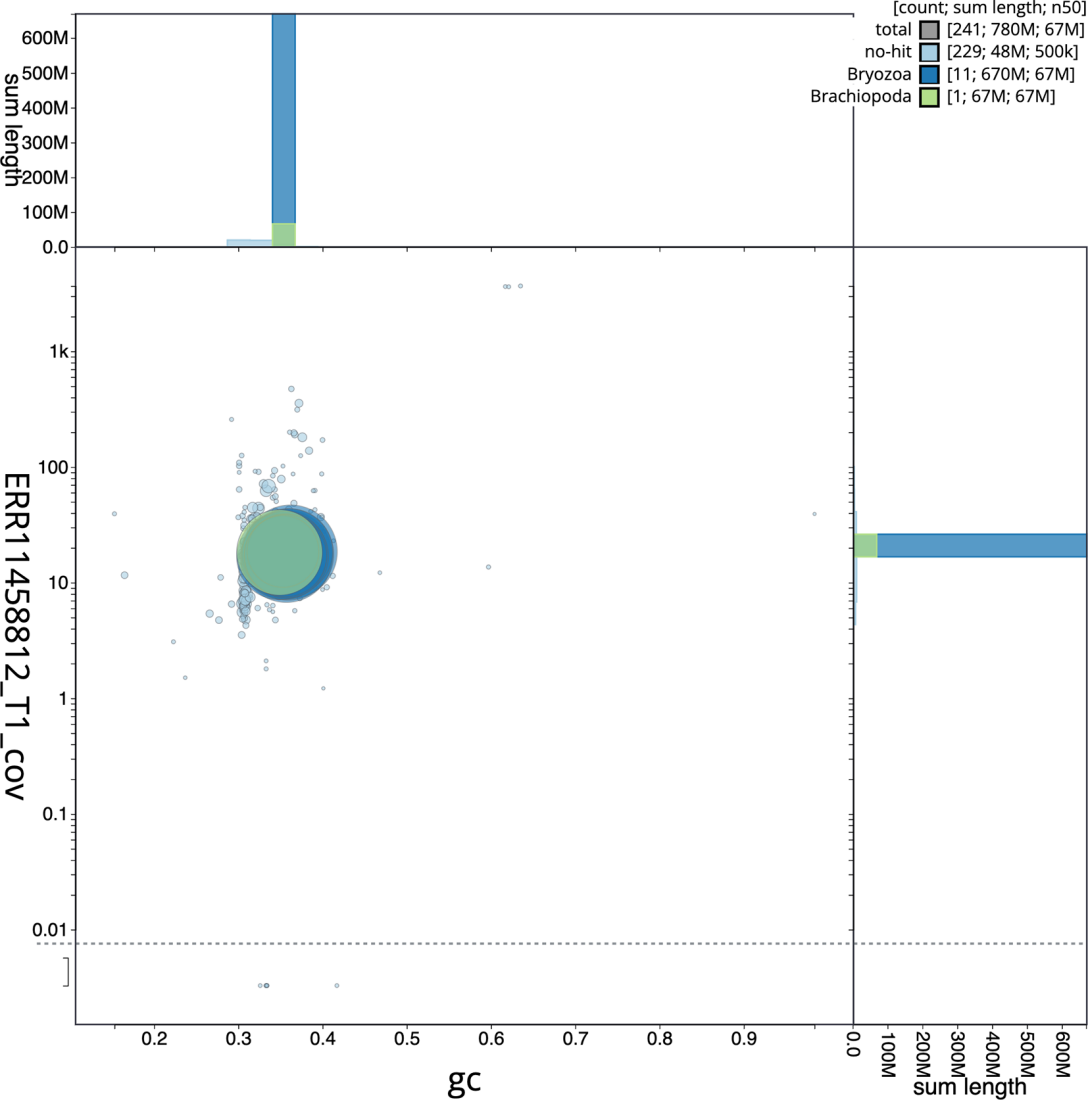
Genome assembly of *Watersipora subatra*, tzWatSuba1.1: BlobToolKit GC-coverage plot. Sequences are coloured by phylum. Circles are sized in proportion to sequence length. Histograms show the distribution of sequence length sum along each axis. An interactive version of this figure is available at https://blobtoolkit.genomehubs.org/view/Watersipora_subatra/dataset/GCA_963576615.1/blob.

**Figure 4.**
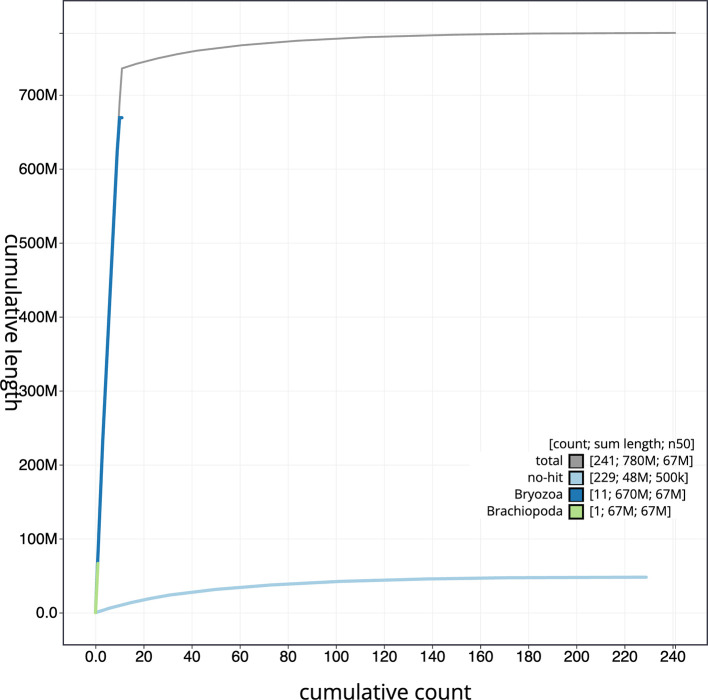
Genome assembly of *Watersipora subatra* tzWatSuba1.1: BlobToolKit cumulative sequence plot. The grey line shows cumulative length for all sequences. Coloured lines show cumulative lengths of sequences assigned to each phylum using the buscogenes taxrule. An interactive version of this figure is available at https://blobtoolkit.genomehubs.org/view/Watersipora_subatra/dataset/GCA_963576615.1/cumulative.

**Figure 5.**
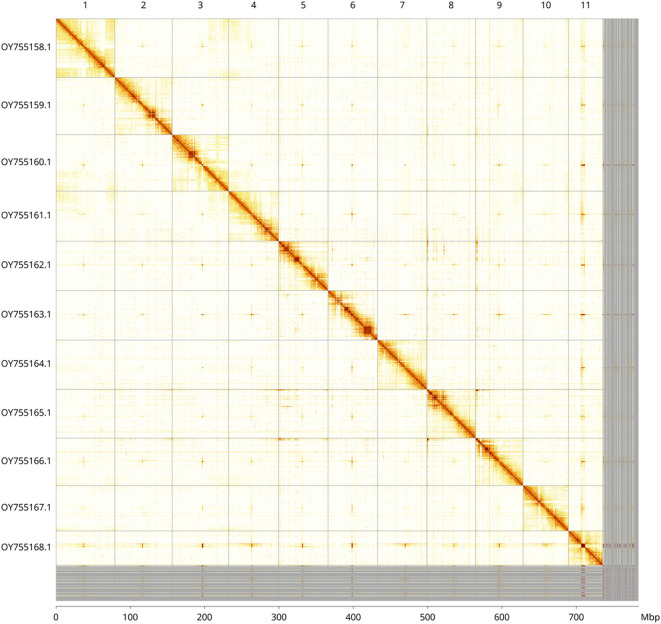
Genome assembly of *Watersipora subatra* tzWatSuba1.1: Hi-C contact map of the tzWatSuba1.1 assembly, visualised using PretextSnapshot. Chromosomes are shown in order of size from left to right and top to bottom. An interactive HiGlass version of the contact map may be viewed at https://genome-note-higlass.tol.sanger.ac.uk/l/?d=amyOadGCR8WdktHcCGuL8Q.

Manual assembly curation corrected 174 missing joins or mis-joins and 46 haplotypic duplications, reducing the assembly length by 3.02% and the scaffold number by 13.31%, and increasing the scaffold N50 by 1.28%. The final assembly has a total length of 783.70 Mb in 240 sequence scaffolds with a scaffold N50 of 66.5 Mb ( Table 2). The total count of gaps in the scaffolds is 255. The snail plot in Figure 2 provides a summary of the assembly statistics, while the distribution of assembly scaffolds on GC proportion and coverage is shown in Figure 3. The cumulative assembly plot in Figure 4 shows curves for subsets of scaffolds assigned to different phyla. Most (93.87%) of the assembly sequence was assigned to 11 chromosomal-level scaffolds. Chromosome-scale scaffolds confirmed by the Hi-C data are named in order of size ( Figure 5; Table 3). Some centromeric repeat sequences could not be assigned uniquely to a chromosome. While not fully phased, the assembly deposited is of one haplotype. Contigs corresponding to the second haplotype have also been deposited. The mitochondrial genome was also assembled and can be found as a contig within the multifasta file of the genome submission.

**Table 2.** Genome assembly data for *Watersipora subatra*, tzWatSuba1.1.

Genome assembly	
Assembly name	tzWatSuba1.1
Assembly accession	GCA_963576615.1
*Accession of alternate haplotype*	*GCA_963576605.1*
Span (Mb)	783.70
Number of contigs	496
Contig N50 length (Mb)	4.5
Number of scaffolds	240
Scaffold N50 length (Mb)	66.5
Longest scaffold (Mb)	79.35

Assembly metrics *		* Benchmark*
Consensus quality (QV)	Primary: 63.2; alternate: 63.8; combined: 63.5	≥ *40*
*k*-mer completeness	Primary: 69.65%; alternate: 61.25%; combined: 98.05%	≥ *95%*
BUSCO **	C:83.4%[S:81.7%,D:1.7%],F:8.6%,M:8.0%,n:954	*C* ≥ *95%*
Percentage of assembly mapped to chromosomes	93.87%	≥ *90%*
Sex chromosomes	None	*localised homologous pairs*
Organelles	Mitochondrial genome: 14.14 kb	*complete single alleles*

Genome annotation of assembly GCA_963576615.1 at Ensembl	
Number of protein-coding genes	16,835
Number of non-coding genes	17,199
Number of gene transcripts	43,769

* Assembly metric benchmarks are adapted from column VGP-2020 of “ Table 1: Proposed standards and metrics for defining genome assembly quality” from Rhie *et al.* (2021).

** BUSCO scores based on the metazoa_odb10 BUSCO set using version 5.4.3. C = complete [S = single copy, D = duplicated], F = fragmented, M = missing, n = number of orthologues in comparison. A full set of BUSCO scores is available at https://blobtoolkit.genomehubs.org/view/Watersipora_subatra/dataset/GCA_963576615.1/busco.

**Table 3.** Chromosomal pseudomolecules in the genome assembly of *Watersipora subatra*, tzWatSuba1.

INSDC accession	Name	Length (Mb)	GC%
OY755158.1	1	79.35	36.0
OY755159.1	2	77.13	35.5
OY755160.1	3	75.93	36.0
OY755161.1	4	67.13	36.0
OY755162.1	5	66.62	35.0
OY755163.1	6	66.55	35.0
OY755164.1	7	66.31	35.5
OY755165.1	8	65.55	35.5
OY755166.1	9	64.04	35.5
OY755167.1	10	61.08	35.5
OY755168.1	11	46.06	35.5
OY755169.1	MT	0.01	29.5

The combined primary and alternate assemblies achieve an estimated QV of 63.5. The *k*-mer completeness is 69.65% for the primary assembly, 61.25% for the alternate haplotype, and 98.05% for the combined assemblies. BUSCO v.5.5.0 analysis using the metazoa_odb10 reference set ( *n* = 954) identified 83.4% of the expected gene set (single = 81.7%, duplicated = 1.7%).

## Genome annotation report

The *Watersipora subatra* genome assembly (GCA_963576615.1) was annotated by Ensembl at the European Bioinformatics Institute (EBI). This annotation includes 43,769 transcribed mRNAs from 16,835 protein-coding and 17,199 non-coding genes. The average transcript length is 10,467.79 bp, with an average of 1.29 coding transcripts per gene and 5.52 exons per transcript. The annotation files may be downloaded from the Ensembl annotation page.

## Methods

### Sample acquisition

The specimen used for genome sequencing was an adult *Watersipora subatra* (specimen ID MBA-191001-001A, ToLID tzWatSuba1) was collected by hand from a fender buoy on King Point Marina, Plymouth, England, UK (latitude 50.37, longitude –4.15) on 2019-10-01. The specimen used for Hi-C sequencing (specimen ID MBA-201019-018C, ToLID tzWatSuba6) was an adult specimen collected by hand from the Torquay Marina, Devon, England, UK (latitude 50.46, longitude –3.53) on 2020-10-19. The specimen used for RNA sequencing (specimen ID MBA-220119-006A, ToLID tzWatSuba4) was an adult specimen collected from Queen Anne’s Battery, Plymouth, England, UK (latitude 50.36, longitude –4.13) on 2022-01-19. The specimens were collected by John Bishop and Christine Wood and identified by John Bishop.

### Nucleic acid extraction

The workflow for high molecular weight (HMW) DNA extraction at the WSI Tree of Life Core Laboratory includes a sequence of core procedures: sample preparation; sample homogenisation, DNA extraction, fragmentation, and clean-up. In sample preparation, the tzWatSuba1 sample was weighed and dissected on dry ice ( Jay *et al.*, 2023). Tissue was homogenised using a PowerMasher II tissue disruptor ( Denton *et al.*, 2023a).

HMW DNA was extracted using the Manual MagAttract v1 protocol ( Strickland *et al.*, 2023b). DNA was sheared into an average fragment size of 12–20 kb in a Megaruptor 3 system ( Todorovic *et al.*, 2023). Sheared DNA was purified by solid-phase reversible immobilisation ( Strickland *et al.*, 2023a): in brief, the method employs AMPure PB beads to eliminate shorter fragments and concentrate the DNA. The concentration of the sheared and purified DNA was assessed using a Nanodrop spectrophotometer and Qubit Fluorometer using the Qubit dsDNA High Sensitivity Assay kit. Fragment size distribution was evaluated by running the sample on the FemtoPulse system.

RNA was extracted from tzWatSuba4 in the Tree of Life Laboratory at the WSI using the RNA Extraction: Automated MagMax™ *mir*Vana protocol ( do Amaral *et al.*, 2023). The RNA concentration was assessed using a Nanodrop spectrophotometer and a Qubit Fluorometer using the Qubit RNA Broad-Range Assay kit. Analysis of the integrity of the RNA was done using the Agilent RNA 6000 Pico Kit and Eukaryotic Total RNA assay.

Protocols developed by the WSI Tree of Life laboratory are publicly available on protocols.io ( Denton *et al.*, 2023b).

### Sequencing

Pacific Biosciences HiFi circular consensus DNA sequencing libraries were constructed according to the manufacturers’ instructions. Poly(A) RNA-Seq libraries were constructed using the NEB Ultra II RNA Library Prep kit. DNA and RNA sequencing was performed by the Scientific Operations core at the WSI on Pacific Biosciences Sequel II (HiFi) and Illumina NovaSeq 6000 (RNA-Seq) instruments. Hi-C data were also generated from tissue of tzWatSuba6 using the Arima-HiC v2 kit. The Hi-C sequencing was performed using paired-end sequencing with a read length of 150 bp on the Illumina NovaSeq 6000 instrument.

### Genome assembly, curation and evaluation

***Assembly***

The original assembly of HiFi reads was performed using Hifiasm ( Cheng *et al.*, 2021) with the --primary option. Haplotypic duplications were identified and removed with purge_dups ( Guan *et al.*, 2020). Hi-C reads are further mapped with bwa-mem2 ( Vasimuddin *et al.*, 2019) to the primary contigs, which are further scaffolded using the provided Hi-C data ( Rao *et al.*, 2014) in YaHS ( Zhou *et al.*, 2023) using the --break option. Scaffolded assemblies are evaluated using Gfastats ( Formenti *et al.*, 2022), BUSCO ( Manni *et al.*, 2021) and Merqury.FK ( Rhie *et al.*, 2020).

The mitochondrial genome was assembled using MitoHiFi ( Uliano-Silva *et al.*, 2023), which runs MitoFinder ( Allio *et al.*, 2020) and uses these annotations to select the final mitochondrial contig and to ensure the general quality of the sequence.

***Assembly curation***

The assembly was decontaminated using the Assembly Screen for Cobionts and Contaminants (ASCC) pipeline (article in preparation). Flat files and maps used in curation were generated in TreeVal ( Pointon *et al.*, 2023). Manual curation was primarily conducted using PretextView ( Harry, 2022), with additional insights provided by JBrowse2 ( Diesh *et al.*, 2023) and HiGlass ( Kerpedjiev *et al.*, 2018). Scaffolds were visually inspected and corrected as described by Howe *et al*. (2021). Any identified contamination, missed joins, and mis-joins were corrected, and duplicate sequences were tagged and removed. The entire process is documented at https://gitlab.com/wtsi-grit/rapid-curation (article in preparation).

***Evaluation of the final assembly***

The Merqury.FK tool ( Rhie *et al.*, 2020) was run in a Singularity container ( Kurtzer *et al.*, 2017) to evaluate *k*-mer completeness and assembly quality for the primary and alternate haplotypes using the *k*-mer databases ( *k* = 31) computed prior to genome assembly. The analysis outputs included assembly QV scores and completeness statistics.

The genome was analysed using the BlobToolKit pipeline, a Nextflow implementation of the earlier Snakemake version ( Challis *et al.*, 2020). The pipeline aligns PacBio reads using minimap2 ( Li, 2018) and SAMtools ( Danecek *et al.*, 2021) to generate coverage tracks. It runs BUSCO ( Manni *et al.*, 2021) using lineages identified from the NCBI Taxonomy ( Schoch *et al.*, 2020). For the three domain-level lineages, BUSCO genes are aligned to the UniProt Reference Proteomes database ( Bateman *et al.*, 2023) using DIAMOND blastp ( Buchfink *et al.*, 2021). The genome is divided into chunks based on the density of BUSCO genes from the closest taxonomic lineage, and each chunk is aligned to the UniProt Reference Proteomes database with DIAMOND blastx. Sequences without hits are chunked using seqtk and aligned to the NT database with blastn ( Altschul *et al.*, 1990). The BlobToolKit suite consolidates all outputs into a blobdir for visualisation. The BlobToolKit pipeline was developed using nf-core tooling ( Ewels *et al.*, 2020) and MultiQC ( Ewels *et al.*, 2016), with containerisation through Docker ( Merkel, 2014) and Singularity ( Kurtzer *et al.*, 2017).

Table 4 contains a list of relevant software tool versions and sources.

**Table 4.** Software tools: versions and sources.

Software tool	Version	Source
BEDTools	2.30.0	https://github.com/arq5x/bedtools2
BLAST	2.14.0	ftp://ftp.ncbi.nlm.nih.gov/blast/executables/blast+/
BlobToolKit	4.3.7	https://github.com/blobtoolkit/blobtoolkit
BUSCO	5.5.0	https://gitlab.com/ezlab/busco
bwa-mem2	2.2.1	https://github.com/bwa-mem2/bwa-mem2
Cooler	0.8.11	https://github.com/open2c/cooler
DIAMOND	2.1.8	https://github.com/bbuchfink/diamond
fasta_windows	0.2.4	https://github.com/tolkit/fasta_windows
FastK	1.1	https://github.com/thegenemyers/FASTK
Gfastats	1.3.6	https://github.com/vgl-hub/gfastats
GoaT CLI	0.2.5	https://github.com/genomehubs/goat-cli
Hifiasm	0.16.1-r375	https://github.com/chhylp123/hifiasm
HiGlass	1.13.4	https://github.com/higlass/higlass
Merqury.FK	1.1.2	https://github.com/thegenemyers/MERQURY.FK
Minimap2	2.24-r1122	https://github.com/lh3/minimap2
MitoHiFi	3	https://github.com/marcelauliano/MitoHiFi
MultiQC	1.14, 1.17, and 1.18	https://github.com/MultiQC/MultiQC
NCBI Datasets	15.12.0	https://github.com/ncbi/datasets
Nextflow	23.04.0-5857	https://github.com/nextflow-io/nextflow
PretextSnapshot	0.0.5	https://github.com/sanger-tol/PretextSnapshot
PretextView	0.2.5	https://github.com/sanger-tol/PretextView
purge_dups	1.2.5	https://github.com/dfguan/purge_dups
samtools	1.18	https://github.com/samtools/samtools
sanger-tol/ascc	-	https://github.com/sanger-tol/ascc
sanger-tol/curationpretext	1.4.2	https://github.com/sanger-tol/curationpretext
Seqtk	1.3	https://github.com/lh3/seqtk
Singularity	3.9.0	https://github.com/sylabs/singularity
TreeVal	1.0.0	https://github.com/sanger-tol/treeval
YaHS	1.2a.2	https://github.com/c-zhou/yahs

### Genome annotation

The Ensembl Genebuild annotation system ( Aken *et al.*, 2016) was used to generate annotation for the *Watersipora subatra* assembly (GCA_963576615.1) in Ensembl Rapid Release at the EBI. Annotation was created primarily through alignment of transcriptomic data to the genome, with gap filling via protein-to-genome alignments of a select set of proteins fromUniProt ( UniProt Consortium, 2019).

### Wellcome Sanger Institute – Legal and Governance

The materials that have contributed to this genome note have been supplied by a Darwin Tree of Life Partner. The submission of materials by a Darwin Tree of Life Partner is subject to the ‘Darwin Tree of Life Project Sampling Code of Practice’, which can be found in full on the Darwin Tree of Life website here. By agreeing with and signing up to the Sampling Code of Practice, the Darwin Tree of Life Partner agrees they will meet the legal and ethical requirements and standards set out within this document in respect of all samples acquired for, and supplied to, the Darwin Tree of Life Project.

Further, the Wellcome Sanger Institute employs a process whereby due diligence is carried out proportionate to the nature of the materials themselves, and the circumstances under which they have been/are to be collected and provided for use. The purpose of this is to address and mitigate any potential legal and/or ethical implications of receipt and use of the materials as part of the research project, and to ensure that in doing so we align with best practice wherever possible. The overarching areas of consideration are:

• Ethical review of provenance and sourcing of the material
• Legality of collection, transfer and use (national and international)

Each transfer of samples is further undertaken according to a Research Collaboration Agreement or Material Transfer Agreement entered into by the Darwin Tree of Life Partner, Genome Research Limited (operating as the Wellcome Sanger Institute), and in some circumstances other Darwin Tree of Life collaborators.

## Author information

Members of the Marine Biological Association Genome Acquisition Lab are listed here: https://doi.org/10.5281/zenodo.8382513.

Members of the Darwin Tree of Life Barcoding collective are listed here: https://doi.org/10.5281/zenodo.12158331

Members of the Wellcome Sanger Institute Tree of Life Management, Samples and Laboratory team are listed here: https://doi.org/10.5281/zenodo.12162482.

Members of Wellcome Sanger Institute Scientific Operations: Sequencing Operations are listed here: https://doi.org/10.5281/zenodo.12165051.

Members of the Wellcome Sanger Institute Tree of Life Core Informatics team are listed here: https://doi.org/10.5281/zenodo.12160324.

Members of the Tree of Life Core Informatics collective are listed here: https://doi.org/10.5281/zenodo.12205391.

Members of the Darwin Tree of Life Consortium are listed here: https://doi.org/10.5281/zenodo.4783558.

## Data Availability

European Nucleotide Archive: *Watersipora subatra* (red ripple bryozoan). Accession number PRJEB62167; https://identifiers.org/ena.embl/PRJEB62167. The genome sequence is released openly for reuse. The *Watersipora subatra* genome sequencing initiative is part of the Darwin Tree of Life (DToL) project. All raw sequence data and the assembly have been deposited in INSDC databases. Raw data and assembly accession identifiers are reported in Table 1 and Table 2. Production code used in genome assembly at the WSI Tree of Life is available at https://github.com/sanger-tol .
