# Supplementary material for: The genome sequence of the red ripple bryozoan, Watersipora subatra (Ortmann, 1890)
Source: Wellcome Open Res. 2026 Feb 11;9:458. Originally published 2024 Aug 12. [Version 2] doi: 10.12688/wellcomeopenres.22824.2 (PMC12902671; doi:10.12688/wellcomeopenres.22824.2)
Supplement: Peer review history

# Reviewer report: Reviewer response for version 2

**DOI:** 10.21956/wellcomeopenres.28650.r147403

**Published:** 2026-02-12

**Authors:** Corinna Breusing

**Affiliations:** 1 University of Rhode Island, Narragansett, Rhode Island, USA

I would like to thank the authors for addressing my concerns and including my suggestions into their revisions. I am satisfied with the authors’ responses and do not have further suggestions.

Are sufficient details of methods and materials provided to allow replication by others?

Yes

Is the rationale for creating the dataset(s) clearly described?

Partly

Are the datasets clearly presented in a useable and accessible format?

Yes

Are the protocols appropriate and is the work technically sound?

Yes

Reviewer Expertise:

NA

I confirm that I have read this submission and believe that I have an appropriate level of expertise to confirm that it is of an acceptable scientific standard.

# Reviewer report: Reviewer response for version 1

**DOI:** 10.21956/wellcomeopenres.25131.r98325

**Published:** 2024-09-25

**Authors:** Lee Hsiang Liow

**Affiliations:** 1 Natural History Museum, University of Oslo, Oslo, Norway

Bryozoans are an ecologically and evolutionarily important Phylum that has received disproportionately little attention from the molecular phylogenetics and other biological fields of study, hence this work is greatly appreciated. Bishop and Wood present data from a cheilostome bryozoan they sequenced in a systematic and transparent way.

**Comments:**

- Have other cheilostome bryozoans been sequenced by ToL? Might be good idea to list the handful of whole genomes sequenced/available to date and to explain in a couple of sentences their general importance (see e.g. Orr et al. 2022 Sci Adv). This will give a solid justification (in addition to helping with taxonomic issues) for why genome sequences of cheilostomes is needed.
- Do I understand correctly that other than Fig. 1, no remaining physical or digital vouchers of  the sequenced specimens remain? If they do, please give the accession numbers. Because *Watertspiora *is such a problematic genus and because light photographs with little detail are difficult to impossible for morphologists to work with, physical vouchers or at least scanning electron micrographs of fragments are always recommended for bryozoan sequencing projects. E.g. see Orr et al. 2022 Paleozoic origins of cheilostome bryozoans and parental care inferred by a new genome-skimmed phylogeny. Science Advances 8(13) https://www.science.org/doi/10.1126/sciadv.abm7452 for their digital vouchers. In Fig. 1, please include scale bars.
- In Methods, it might be good to state that they are hermaphrodite adults.
- Figure 2: I guess this is a standard output, but I am not sure what Scale (bottom left) means. Please list the short forms used (for BUSCO) in the legend. And for the left bottom, what is N? Not assignable to ATCG?|
- Figure 3: Just my ignorance for what might be standard in the online too., but where does the brachiopod data come from and is it for comparison to the bryozoans (so is the blue referring to the *Watersipora* you sequenced?) Is it possible to improve the axis labels? Right now, as an educated biologist but not a molecular one or bioinformatician, I can only guess what the axes mean (GC content on x axis?The other chunks are mysterious, so are the size of the blobs, please explain).
- Figure 4: same issue with the brachiopod as above. No-hit compared to what?
- Figure 5: what do the colors mean, and might it be possible to label the chromosomes e.g. Table 3 numbering?
- Bioinformatic tools table: great that the software and versions are listed. What about the computational platforms used for the current analyses?

Are sufficient details of methods and materials provided to allow replication by others?

Yes

Is the rationale for creating the dataset(s) clearly described?

Partly

Are the datasets clearly presented in a useable and accessible format?

Partly

Are the protocols appropriate and is the work technically sound?

Yes

Reviewer Expertise:

I am a paleobiologist and macroevolutionary biologist who has contributed to the sampling and sequencing of bryozoans (funding, data acquisition, curation, data analyses, paper writing).

I confirm that I have read this submission and believe that I have an appropriate level of expertise to confirm that it is of an acceptable scientific standard, however I have significant reservations, as outlined above.

## References

[1] : Paleozoic origins of cheilostome bryozoans and their parental care inferred by a new genome-skimmed phylogeny. *Sci Adv* .2022;8(13) : 10.1126/sciadv.abm7452 eabm7452 10.1126/sciadv.abm7452 35353568 PMC8967238

# Reviewer report: Reviewer response for version 1

**DOI:** 10.21956/wellcomeopenres.25131.r98332

**Published:** 2024-09-22

**Authors:** Nickellaus Roberts

**Affiliations:** 1 Department of Biological Sciences, The University of Alabama, Tuscaloosa, USA

Bishop and Wood describe the genome sequencing and assembly of the red ripple bryozoan *Watersipora subatra. *The authors use methods that are clear and represent the industry standard. The presented datasets are clear and accessible, and all genome metrics are presented in accordance of what is expected for a genome data note. Scripts to reproduce the assembly can be found from https://github.com/sanger-tol/genomenote, which has good documentation.

**Minor Comments:**

- The BUSCO score may concern readers who are not familiar with BUSCO and invertebrates. Authors report a completeness score of 83.4% which is perfectly acceptable for an organism in this clade. It may be useful to report BUSCO scores from other bryozoans to clarify this.
- I understand that incorporation of different Hi-C tools in these pipelines (used for many projects) may be difficult, but HapHiC performs better if not just as well as YAHS, and moving towards more haplotype resolved assemblies (that HapHiC can help provide, without a reference) would be great to see from the Welcome Sanger Team. 

**Major Comments:**

- Please specify that the BUSCO completeness is nucleotide, and please report the the protein BUSCO score for annotation.

Are sufficient details of methods and materials provided to allow replication by others?

Partly

Is the rationale for creating the dataset(s) clearly described?

Yes

Are the datasets clearly presented in a useable and accessible format?

Yes

Are the protocols appropriate and is the work technically sound?

Yes

Reviewer Expertise:

genomics, phylogenomics, low-input genomics, kamptozoa, phylogenetics, systematics

I confirm that I have read this submission and believe that I have an appropriate level of expertise to confirm that it is of an acceptable scientific standard, however I have significant reservations, as outlined above.

## References

[1] : Chromosome-level scaffolding of haplotype-resolved assemblies using Hi-C data without reference genomes. *Nat Plants* .2024;10(8) : 10.1038/s41477-024-01755-3 1184-1200 10.1038/s41477-024-01755-3 39103456

[2] sanger-tol/genomenote. Reference source

# Reviewer report: Reviewer response for version 1

**DOI:** 10.21956/wellcomeopenres.25131.r98329

**Published:** 2024-09-18

**Authors:** Corinna Breusing

**Affiliations:** 1 University of Rhode Island, Narragansett, Rhode Island, USA

The data note by Bishop & Wood describes the genome sequence of the red ripple bryozoan *Watersipora subatra*. The authors use established, state-of-the-art methods from the Tree of Life Consortium for sequencing, assembly, annotation and curation. All protocols are well documented, while the datasets are clearly presented and describe the main results in an accessible way.

**There are only a couple of points I found that could be improved:**

 •    While the introduction provides sufficient background information about the study organism, the rationale for creating the genomic dataset could be made a little clearer. The authors mention that there is a lot of confusion about the taxonomy in the *Watersipora* genus, which I assume is one of the reasons for sequencing the genome of *W. subatra*. Are there plans for sequencing additional *Watersipora* genomes (in particular *W. subtorquata*) to better delineate species within the genus, for example to help monitor their spread and invasion to other regions? I think some of these details could clarify the reasoning for sequencing the genome of *W. subatra*.

 •    For full reproducibility it might be helpful to upload the bioinformatic scripts that outline the exact command lines for the different analysis steps to a separate GitHub repository

Are sufficient details of methods and materials provided to allow replication by others?

Yes

Is the rationale for creating the dataset(s) clearly described?

Partly

Are the datasets clearly presented in a useable and accessible format?

Yes

Are the protocols appropriate and is the work technically sound?

Yes

Reviewer Expertise:

Bioinformatics, deep-sea biology, symbiosis, genomics

I confirm that I have read this submission and believe that I have an appropriate level of expertise to confirm that it is of an acceptable scientific standard, however I have significant reservations, as outlined above.

# Response

**Published:** 2026-02-04

**Authors:** Tree of Life Team Sanger

**Affiliations:** Wellcome Trust Sanger Institute, UK

In response to the first point, we have clarified the rationale for generating this genome assembly in the Introduction. The sequencing of *Watersipora subatra* forms part of the Darwin Tree of Life (DToL) project, which aims to generate reference genome assemblies across the breadth of UK biodiversity. The primary objective of the project is to provide high-quality genome sequence resources rather than to resolve taxonomic questions or to target specific species complexes. While genomic resources may support future taxonomic and ecological studies, including work on species delineation within *Watersipora*, the selection of species for sequencing depends on specimen availability through project partners. In response to the second point, we have expanded the Data availability section to state that production code used for genome assembly within the Wellcome Sanger Institute Tree of Life programme is publicly available at https://github.com/sanger-tol. This repository contains the maintained pipelines and workflows used for assembly, curation and evaluation, providing transparency and reproducibility at the level of production analyses.

 The Tree of Life team, Wellcome Sanger Institute
